# Impact of PD-1 and PD-L1 expression on treatment outcomes in newly diagnosed acute myeloid leukemia patients

**DOI:** 10.1016/j.lrr.2025.100514

**Published:** 2025-05-09

**Authors:** Ugur Calis, Merve Aydogan, Guldane Cengiz Seval, Klara Dalva, Selami Kocak Toprak

**Affiliations:** a Department of Internal Medicine, Ankara University, Ankara, Turkey; b Department of Hematology, Ankara University, Ankara, Turkey

**Keywords:** Acute myeloid Leukemia, Aml, PD-1, PD-L1, Flow cytometry

## Abstract

High expression of immune checkpoint markers may leukemic cells to evade the immune system in AML. This study aimed to investigate the relationship between PD-1/PD-L1 expression and treatment outcomes in AML patients.. The study included 21 patients and 18 healthy volunteers. Non-responders exhibited significantly higher PD-1 expression (MFI) in CD3+ and CD4+ *T* cells. At the time of diagnosis, bone marrow samples from patients exhibited a significantly higher proportion of PD-1 expression in CD3+, CD4+, and CD8+ *T* lymphocytes than peripheral blood samples. The results revealed an association between PD-1/PD-L1 expression and clinical traits in newly diagnosed AML patients.

## 1 Introduction

Acute myeloid leukemia (AML) is a malignant disorder of the hematopoietic system characterized by rapid proliferation and accumulation of immature myeloid cells in the bone marrow, which eventually hinders the production of normal blood cells [1]. Despite the use of traditional chemotherapy and targeted therapies to treat this highly heterogeneous disease, the mortality rates remain high.

The expression of PD-1 and PD-L1 has been extensively studied in various malignancies, and their correlation with prognosis has been established in several cancer types [2]. In acute myeloid leukemia, elevated expression of immune checkpoint molecules generates an immunosuppressive tumor microenvironment and is linked to disease progression. The connection between PD-1 and PD-L1 promotes T cell death, suppresses T cell proliferation, and prevents cytokine release, all of which help tumor cells evade the immune system [3].

A comprehensive study that included 176 AML patients from TCGA database and 62 more patients whose bone marrow samples were confirmed revealed a correlation between low overall survival and high expression levels of PD-1, PD-L1, and PD-L2, demonstrating the potential of these markers as prognostic indicators [4]. Huang et al. demonstrated a significantly increased distribution of PD-1 + Vβ+ *T* cells in AML patients compared to healthy individuals, suggesting a potential association between poor prognosis and reduced anti-leukemia effects [5]. The study highlights that the expression of PD-L1, PD-L2, PD-1, and CTLA4 is upregulated in myelodysplastic syndromes (MDS), chronic myelomonocytic leukemia (CMML), and acute myeloid leukemia (AML) patients, and is further enhanced by treatment with hypomethylating agents, suggesting a potential role in disease pathogenesis and resistance to therapy [6]. Previous studies have emphasized the importance of immune checkpoint markers in acute myeloid leukemia. The higher expression of PD-1 in patients with AML than that in healthy controls may explain the use of this marker as a therapeutic target. Detailed evaluation of these markers and their relationship with the disease may be important in terms of both prognosis and treatment.

In this study, we aimed to investigate the relationship between PD-1/PD-L1 expression and prognosis in patients with AML. By analyzing the expression levels of these immune checkpoint molecules and their correlation with clinical outcomes, we aimed to expand the corpus of knowledge regarding immunotherapy targets in AML. Our findings may have a significant impact on therapy choices, risk assessment, and the eventual enhancement of AML patient outcomes.

## 2 Methods

Patients diagnosed with de novo AML between April 2023 and November 2023 in the Department of Hematology at Ankara University Faculty of Medicine were included in this study.

### 2.1 Sample collection

PD-1 expression in T cells and PD-L1 expression in leukemic blasts were determined using flow cytometry in bone marrow aspirate samples at the time of diagnosis. After remission induction and consolidation therapy, PD-1 levels were re-evaluated in the bone marrow aspiration samples of the patients.

When the patients were first diagnosed, PD-1 expression levels in their peripheral blood samples were measured and compared with PD-1 levels in their bone marrow samples. The levels of PD-1 expression in the peripheral blood samples taken from the patients at the time of diagnosis were compared to those of age- and sex-matched healthy volunteers.

Bone marrow and peripheral blood samples were collected before the start of leukemia treatment. All patients and healthy volunteers were informed of the study and signed an informed consent form before the collection and study.

### 2.2 Flow cytometric analysis

Peripheral blood samples and bone marrow aspirate samples were collected in EDTA tubes from each patient in the experimental group at the time of diagnosis upon inclusion in the study. Bone marrow aspirate samples were collected at the end of the induction therapy and consolidation therapy. These samples were transferred to the hematology laboratory under appropriate conditions, with tubes maintained horizontally at room temperature, and delivered to the laboratory within a maximum of 4 h after collection. Peripheral blood samples from each volunteer in the control group were collected and transferred to the hematology laboratory under the appropriate conditions.

PD-1 expression in T cells and PD-L1 expression in leukemic blasts were determined using flow cytometry, utilizing monoclonal antibodies against the CD3, CD4, CD8, CD45, CD38, CD34, CD117, HLA-DR, CD279 (PD-1), and CD274 (PD-L1) surface markers. Additionally, the intensity of PD-1 and PD-L1 expression in cells was evaluated by recording mean fluorescence intensity (MFI).

Multiparameter flow cytometry was used for measurable residual disease (MRD) assessment, with an evaluation sensitivity of 10⁻⁵.

The antibodies and fluorochromes used are summarized in Table 1.

**Table 1:** The antibodies and fluorochromes used in the study.

**Antibody**	**Fluorochrome**
CD45	KO
CD3	FITC
CD4	ECD
CD8	A700
CD38	A750
CD34	PE
CD117	PC7
HLA-DR	PB
CD274(PD-L1)	APC
CD279(PD-1)	PC5.5

### 2.3 Response evaluation

Patients diagnosed with de novo AML received remission induction chemotherapy according to protocols determined by the attending physician, following evaluation of PD-1 and PD-L1 expression status at the time of diagnosis. Response evaluation was performed within 28 days of treatment initiation using bone marrow aspiration and biopsy. The response assessment criteria were based on the ELN 2022 guidelines, and the patients were grouped accordingly.

### 2.4 Statistical analysis

Normally distributed quantitative variables are presented as mean ± standard deviation and assessed using the *t*-test, while skewed data are described as median (minimum-maximum) and compared using the Mann-Whitney U test. Nominal variables are shown as frequencies (%) and assessed for significance using Pearson’s chi-squared test or Fisher’s exact test between the two groups. The normality of the data was evaluated using the Shapiro-Wilk and Kolmogorov-Smirnov tests.

The discrimination of PD-1 expression levels according to their response to treatment was examined using the area under the curve (AUC) and the process characteristic curve (ROC), and the cut-off points for these parameters were taken as the point where the Youden Index (*J*= Sensitivity + Selectivity −1) was maximum. For the data classified according to these cut-off points, Kaplan-Meier analysis was performed for survival times and the groups were compared using the log-rank test.

The relationship between continuous variables was assessed using the Pearson correlation test when the distribution was normal and the Spearman correlation test when the distribution was skewed. Some data were visualized using means and 95 % confidence intervals.

All statistical analyses were performed using the IBM SPSS version 25.0 software (SPSS Inc., Chicago, IL, USA), with statistical significance set at *p* < 0,05.

## 3 Results

### 3.1 Patient characteristics

This study included 26 patients and 23 healthy controls (HCs). The median patient age was 45 [20−77]. AML was diagnosed in 8 males and 18 females.

Upon analyzing the FAB subtypes of the patients, two were identified as M0, nine as M2, two as M3, eight as M4, four as M5, and one as M6.

The patients were categorized into risk groups based on the ELN 2022 guidelines. There were 12 patients in the favorable risk group, 12 in the intermediate risk group, and 2 in the adverse risk group.

Based on the ELN 2022 criteria, 9 patients had CR, 4 patients had CRi, 3 had MLFS, 1 had PR, and 4 had no response. Five patients died during induction therapy and, therefore, could not be evaluated for response. For statistical analysis, patients in the CR, CRi, and MLFS groups were considered responders, and patients in the PR and non-responder groups were considered non-responders. Thus, 15 patients were found to be responders to induction therapy and 6 patients were found to be non-responders.

After induction therapy, measurable residual disease (MRD) status was evaluated using flow cytometry. Seven patients were MRD-negative and nine were MRD-positive.

Patient characteristics are shown in Table 2.

**Table 2:** Patient charachteristics.

**Parameter**	**Median (range)**
Age (years)	45 (20–77)
**Parameter**	**Mean ± Standart Deviation (Min-Max)**
Leukocyte count (x10^3^/mm^3^)	43.98 ± 77.13 (2.12–337.67)
Platelet count (x10^3^/mm^3^)	68.27 ± 52.04 (2.00–231.00)
Hemoglobin (g/dL)	9.2 ± 1.5 (7.0–13.8)
LDH (U/L)	549 ± 431 (173–1746)
**Parameter**	**Number (percentage)**
Sex	
Male	8 (%30.8)
Female	18 (%69.2)
FAB Subtypes	
M0	2 (%7.7)
M1	0 (%0)
M2	9 (%34.6)
M3	2 (%7.7)
M4	8 (%30.8)
M5	4 (%15.4)
M6	1 (%3.8)
Genetics	
t(15;17)	2 (%7.7)
NPM1-A	5 (%19.2)
FLT3-TKD	3 (%11.5)
NPM1-*A* + FLT3-ITD/FLT3-TKD	5 (%19.2)
inv(16)	4 (%15.4)
t(8;21)	1 (%3.8)
CEBPA	1 (%3.8)
No genetic characteristics	5 (%19.2)
ELN 2022 Risk Group	
Favorable	12 (%46.2)
Intermediate	13 (%50.0)
Adverse	1 (%3.8)
Response to Induction Therapy	
CR	9 (%35)
CRi	4 (%15)
MLFS	3 (%12)
PR	1 (%4)
No response	4 (%15)
Nonevaluable for response	5 (%19)
Response to Induction Therapy	
Responder	15 (%71.4)
Non-responder	6 (%28.6)
MRD After Induction Therapy	
Negative	7 (%43.8)
Positive	9 (%56.3)

### 3.2 PD-1 expression on T cells

PD-1 expression percentage (%) and intensity (MFI) were evaluated separately in CD3+, CD4+, and CD8+ *T* lymphocyte subgroups in the bone marrow at the time of diagnosis. Patients who did not respond to induction therapy showed significantly higher PD-1 expression intensity (MFI) in CD3+ and CD4+ *T* cells (CD3+: median 3.33 vs 2.73, *p* = 0.008 and CD4+: median 2.97 vs. 2.16, *p* = 0.046, respectively). In CD8+ *T* cells, the PD-1 expression intensity (MFI) was higher in the non-responder group, but the difference was not statistically significant (CD8+: median 4.78 vs. 3.74, *p* = 0.181). The distribution of PD-1 expression levels is presented in Table 3.

**Table 3:** PD-1 expression levels (percentage and MFI) in CD3+, CD4+, and CD8+ *T* lymphocyte subsets, and PD-L1 expression levels (percentage and MFI) in leukemic blasts, according to response to induction therapy.

	Mean ± Standart Deviation	Median (IQR 25–75)	*p*
CD3+ PD-1 Expression Percentage (%)			0.681^a^
Responder	56.3 **±** 14.6	53.7 (46.9–57.9)	
Non-Responder	59.0 **±** 9.54	57.1 (53.9–61.1)	
CD4+ PD-1 Expression Percentage (%)			**0.046^a^**
Responder	54.0 **±** 17.6	49.9 (42.6–60.7)	
Non-Responder	60.4 **±** 16.8	53.8 (50.6–66.6)	
CD8+ PD-1 Expression Percentage (%)			0.454^a^
Responder	61.2 **±** 14.4	57.0 (52.6–63.8)	
Non-Responder	59.7 **±** 11.2	60.6 (55.1–67.0)	
CD3+ PD-1 Expression Intensity (MFI)			**0.008^b^**
Responder	2.92 **±** 0.67	2.73 (2.50–3.04)	
Non-Responder	3.38 ± 0.23	3.33 (3.23–3.38	
CD4+ PD-1 Expression Intensity (MFI)			0.826^a^
Responder	2.31 ± 0.60	2.16 (1.83–2.79)	
Non-Responder	2.89 ± 0.44	2.97 (2.67–3.20)	
CD8+ PD-1 Expression Intensity (MFI)			0.181^a^
Responder	4.25 ± 1.35	3.74 (3.38–5.28)	
Non-Responder	5.21 ± 1.62	4.78 (4.37–5.22)	
Blast PD-L1 Expression Percentage (%)			0.592^a^
Responder	35.3 ± 17.1	33.6 (28.0–40.6)	
Non-Responder	28.1 ± 22.6	23.7 (12.7–31.7)	
Blast PD-L1 Expression Intensity (MFI)			0.340^a^
Responder	1.11 ± 0.16	1.06 (1.02–1.17)	
Non-Responder	1.07 ± 0.11	1.04 (0.99–1.10)	

a: Independent samples *T*-test, b: Mann-Whitney U test, Bold values indicate statistical significance (*p* < 0.05).

**Fig. 1:**
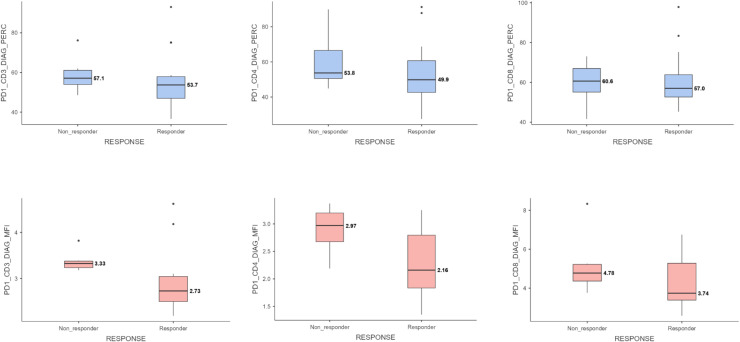
Percentage and intensity (MFI) of PD-1 expression in CD3+, CD4+, and CD8+ *T* lymphocyte subsets at diagnosis according to response to induction therapy. PD-1 expression was significantly higher in CD3+ and CD4+ *T* cells in non-responders. Statistical comparisons were performed using either the Mann–Whitney U test or independent samples *t*-test based on data distribution.

Although the percentage of PD-1 expression on CD3+, CD4+, and CD8+ *T* cells was higher in patients who did not respond to induction therapy, this difference was not statistically significant (*p* = 0.681, *p* = 0.454, and *p* = 0.826, respectively). The corresponding data are presented in Fig. 1.

Using flow cytometry, measurable residual disease (MRD) status was assessed following induction therapy. The intensity of PD-1 expression (MFI) on CD3+, CD4+, and CD8+ *T* cells at the time of diagnosis did not differ significantly between MRD-positive and MRD-negative patient groups. (*p* = 0.844, *p* = 0.306, and *p* = 0.968 respectively). The PD-1 expression percentages of CD3+, CD4+, and CD8+ *T* cells at the time of diagnosis did not differ significantly. (*p* = 0.302, *p* = 0.606 and *p* = 0.371, respectively)

PD-1 expression intensity (MFI) on CD3+ *T* lymphocytes was significantly higher in patients with the AML M5 subtype (*p* = 0.039). PD-1 expression intensity (MFI) in CD3+, CD4+, and CD8+ *T* lymphocytes was higher in patients with NPM1 or FLT3-ITD/FLT3-TKD mutations than in those with other genetic features; however, the difference was not statistically significant (*p* = 0.060, *p* = 0.113, and *p* = 0.300, respectively).

At the time of diagnosis, the intensity of PD-1 expression (MFI) in CD3+ and CD8+ *T* lymphocytes in the bone marrow was higher in patients with CD9-negative leukemic blasts compared to those with CD9-positive leukemic blasts (*p* = 0.017 and *p* = 0.010, respectively). PD-1 expression on CD8+ *T* cells was significantly higher in patients with CD13-positive leukemic blasts compared to those with CD13-negative leukemic blasts (*p* = 0.022). Furthermore, no significant correlation was observed between the expressions of HLA-DR, CD123, CD71, CD64, CD7 and CD56 and the levels of PD-1.

The percentage of PD-1 expression in CD3+, CD4+, and CD8+ *T* lymphocytes in the bone marrow samples obtained from patients at the time of diagnosis was significantly higher than that in the peripheral blood samples (*p* = 0.02, *p* = 0.039, and *p* < 0.001, respectively).

At the time of diagnosis, PD-1 expression intensity (MFI) on CD3+, CD4+, and CD8+ *T* lymphocytes in peripheral blood samples obtained from patients was significantly higher than the intensity of PD-1 expression intensity (MFI) in peripheral blood samples obtained from healthy controls (*p* = 0.001, *p* = 0.004, and *p* < 0.001, respectively). PD-1 expression intensity (MFI) was also higher in CD3+ and CD8+ *T* lymphocytes in peripheral blood samples obtained from patients than in those obtained from healthy controls (*p* = 0.005 and *p* = 0.020, respectively). The percentage of PD-1 expression on CD4+ *T* cells was higher in the patient group, but the difference was not statistically significant (*p* = 0.057).

As the percentage of blasts determined by flow cytometry in peripheral blood samples obtained from patients at the time of diagnosis increased, no correlation was observed between the percentage of PD-1 expression percentage and intensity (MFI) of CD3+, CD4+, and CD8+ *T* lymphocytes in peripheral blood and the blast percentage.

In bone marrow samples collected after induction therapy, PD-1 expression in CD3+ and CD4+ *T* lymphocyte subsets increased both in percentage and intensity (MFI); however, the difference was not statistically significant.

### 3.3 PD-L1 expression on leukemic blasts

**Fig. 2:**
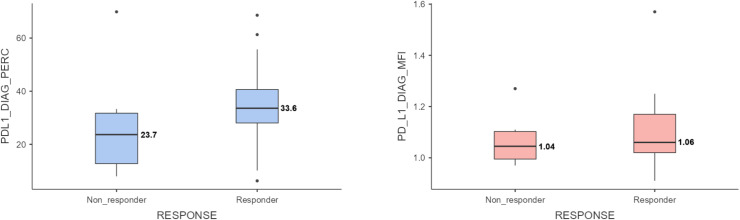
Comparison of PD-L1 expression (percentage and MFI) on leukemic blasts at diagnosis between responders and non-responders to induction therapy. No statistically significant difference was observed between responders and non-responders. Statistical comparisons were performed using either the Mann–Whitney U test or independent samples *t*-test based on data distribution.

No statistically significant correlation was observed between the treatment response, MRD status following induction treatment, and intensity of PD-L1 expression intensity (MFI) in leukemic blast cells at the time of diagnosis. (*p* = 0.592 and *p* = 0.340, respectively). Fig. 2 presents the percentage and intensity (MFI) of PD-L1 expression at diagnosis according to the response to induction therapy. There was no statistically significant relationship among PD-L1 expression percentage, response to induction therapy, and MRD status after induction therapy (*p* = 0.436 and *p* = 0.459, respectively).

At the time of diagnosis, PD-L1 expression intensity (MFI) was significantly higher in patiens with CD7 positive leukemic blasts compared to the CD7 negative. (*p* = 0.014). There was no discernible relationship was observed between PD-L1 expression percentage and intensity (MFI) and HLA-DR, CD13, CD123, CD71, CD64, CD9, or CD56 expression.

There was no statistically significant correlation between leukocyte count, neutrophil count, platelet count, hemoglobin and LDH levels at the time of diagnosis and the percentage and intensity (MFI) of PD-1 and PD-L1 expression percentage and intensity (MFI).

PD-L1 expression in leukemic blasts showed similar levels in bone marrow and peripheral blood, both in percentage (29.50 % vs. 31.42 %; *p* = 0.737) and intensity (MFI: 1.08 vs. 1.07; *p* = 0.754), with no statistically significant differences observed.

**Fig. 3:**
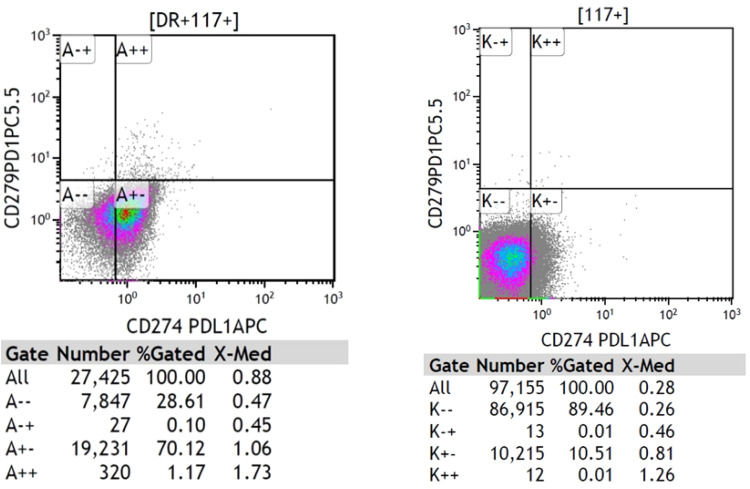
Examples of flow cytometric analysis of patients with high (left) and low (right) PD-L1 expression.

As shown in Fig. 3, examples of flow cytometric analysis of patients with high (left) and low (right) PD-L1 expression are presented.

### 3.4 Survival analysis

For the intensity of PD-1 expression (MFI) at diagnosis in CD3+ and CD4+ *T* lymphocytes, which showed statistical significance according to treatment response, a cut-off value was obtained using the ROC curve according to response status. The cut-off values for PD-1 expression intensity (MFI) in CD3+ and CD4+ *T* lymphocytes were determined as 3.14 and 2.60, respectively. Results below these values were considered "PD-1 negative," and those above these values were considered "PD-1 positive.” In CD3+ *T* lymphocytes, overall survival (OS) was median 8.2 months in the PD-1 positive group and median 11.5 months in the PD-1 negative group; although the difference was clinically significant, it was not statistically significant (*p* = 0.083). For CD4+ *T* lymphocytes, the survival was 8 months in the PD-1 positive group and 12 months in the PD-1 negative group and the difference was statistically significant (*p* = 0.055).

## 4 Discussion

In this study, we evaluated PD-1 expression on T cells and PD-L1 expression on leukemic blasts in the bone marrow of patients diagnosed with AML and its correlation with response to induction therapy, MRD status, genetics, and differences between peripheral blood and bone marrow samples.

Our survival analysis focused on the intensity (MFI) of PD-1 expression in CD3+ and CD4+ *T* lymphocytes at diagnosis and its potential prognostic implications for the overall survival (OS) of patients with AML. Overall survival was longer in PD-1 positive patients with CD3+ and CD4+ *T* lymphocytes. This finding demonstrated clinical significance and approached statistical significance. These findings suggest the possible use of PD-1 expression as a predictive biomarker for AML, particularly in CD4+ *T* cells. Even if the trends are encouraging, more research with larger sample sizes is required to confirm these results and to investigate the underlying mechanisms behind the observed variations in survival outcomes.

In a study by Bassiouny et al., PD-1 levels in 59 newly diagnosed AML patients were measured using flow cytometry at the time of diagnosis. It was found that the patient group with low PD-1 expression reacted to remission induction therapy more frequently when the 28th day response rates following treatment were compared [7]. Twenty-two newly diagnosed AML patients were assessed in a study by Jia et al. The patients were divided into two groups, one with high PD-1 expression and the other with low PD-1 expression, using the mean value as the cut-off. There were no discernible difference between the two groups in terms of OS and responsiveness to induction therapy. This was attributed to the small sample size in the present study [8]. In our study, patients who did not respond to induction therapy exhibited significantly higher mean fluorescence intensity (MFI) of PD-1 on CD3+ and CD4+ *T* cells, suggesting a potential role of PD-1 in mediating T cell dysfunction in non-responders. Although there are conflicting results in the literature, the importance of PD-1 expression in patients with AML needs to be investigated.

In a study that evaluated 53 newly diagnosed AML patients, PD-L1 expression levels were significantly higher in patients refractory to induction therapy [9]. In another study, PD-L1 expression levels were determined by flow cytometry in 40 adult AML patients, and the relationship between PD-L1 expression levels and response to remission induction therapy was investigated. Although PD-L1 expression levels were higher in the group refractory to induction therapy, the difference was not statistically significant [10]. In another study, low PD-L1 expression was found in leukemic blast cells and it was stated that low PD-L1 expression may have a positive prognostic effect and may be associated with higher overall survival [11]. In these studies, PD-L1 expression levels in patients were examined at the time of diagnosis and responses after remission induction therapy were evaluated. In our study, we evaluated the relationship between PD-L1 expression status at the time of diagnosis and the response to induction therapy. No statistically significant relationship was found between PD-L1 expression and response to induction therapy. This was primarily associated with the small number of patients. PD-L1 expression levels in the patients at the time of diagnosis were significantly different. This indicates that PD-L1 may not serve as a reliable biomarker for predicting therapeutic outcomes in AML patients.

Previous studies have not clearly established a relationship between MRD status and immune checkpoint marker levels. To the best of our knowledge, this study is one of the first to explore the relationship between MRD and PD-1/PD-L1 expression status in the current literature. In our study, the MRD status of the patients was evaluated using flow cytometry. In the MRD-positive patient group, both PD-L1 and PD-1 expression levels were higher than those in the MRD-negative patient group; however, no statistically significant difference was observed. As a result of high expression levels of immune checkpoint markers, escape from the immune system may be achieved and the treatment responses of patients may not be deep enough, which may affect prognosis. The increased expression of these markers may have caused MRD positivity and prevented deep responses. The fact that MRD-negative responses were observed in patients with low levels of immune checkpoint marker expression may be interpreted as a stronger T cell-mediated immune response in addition to chemotherapy. Low expression levels of immune checkpoint markers may be an indicator of MRD-negativity.

Chen et al. determined the expression levels of B7-H1 (PD-L1) in 60 AML patients using immunohistochemistry and real-time PCR methods. It was determined that patients with the AML M5 subtype had higher PD-L1 expression than patients with other subtypes. When the total survival rates of patients were evaluated, it was found that patients with high PD-L1 expression had a worse prognosis [12]. In this study, PD-L1 expression levels in leukemia subtypes were evaluated separately, and expression levels were found to be higher in the AML-M5 subtype. In our study, we evaluated the relationship between leukemic subtype, immunologic markers, and prognosis by recording both PD-1 and PD-L1 expression levels and the leukemia subtype of patients. No statistically significant association was found between the FAB subtype and PD-L1 expression. In our study, PD-1 expression intensity (MFI) in CD3+ *T* cells were higher in patients with AML-M5 subtype. With an increase in the number of patients, the difference in expression between the subgroups can be detected more clearly.

The relationship between PD-1 and PD-L1 expression in patients and the mutations and cytogenetic alterations in patients also needs to be investigated. High PD-L1 expression levels in leukemic blasts have been shown to be associated with lower overall survival, especially in patients with FLT3 and NPM1 mutations [13]. In another study, higher PD-L1 expression levels were found in patients with poor cytogenetic risk [14]. Sallman et al. reported that PD-L1 expression was higher in AML and MDS patients with TP53 mutations, which may be effective in the escape of this patient phenotype from the immune system and may be associated with poor prognosis [15]. In our study, we observed that the intensity of PD-1 expression in CD3+, CD4+, and CD8+ *T* lymphocytes was elevated in patients harboring NPM1 or FLT3-ITD/FLT3-TKD mutations than in those with other genetic characteristics. However, these differences were not significant (*p* = 0.060, *p* = 0.113, and *p* = 0.300, respectively). This alignment suggests that while PD-1 expression may be influenced by specific genetic mutations, further research is necessary to fully elucidate the implications of these observations in the context of T cell functionality and immune evasion in AML.

Previous studies have shown that CD7 expression is associated with a worse overall survival rate in patients with AML [16]. A recent retrospective study showed that patientes with CD7 positive blasts were in the adverse risk category according to the ELN criteria and were associated with worse overall survival, which is in line with previous studies [17]. It has been reported that CD7 expression can be found in approximately 30 percent of newly diagnosed AML patients and is maintained in 70 percent of relapses. In our study, significantly higher PD-L1 expression was found in patients with CD7 positive leukemic blasts. This is consistent with the association of CD7 expression with worse risk factors and overall survival rates. Further clarification of the relationship between CD7 and PD-L1 expression will increase the importance of the co-positivity of these two markers as treatment targets and open the door to new therapeutic possibilities. Studies on CD7-targeted CAR-T cells are currently ongoing [18]. The relationship between CD7 and PD-L1 may also play an important role in predicting the response to CAR-T cell therapies targeting CD7 molecule.

CD9 is a surface antigen expressed in approximately 40 percent of acute myeloid leukemia patients with AML [19]. In one study, CD9 was shown to be highly expressed in CD34+CD38- AML leukemic stem cells, but not in normal hematopoietic cells, suggesting that it may be a potential leukemia stem cell marker and therapeutic target [20]. In our study, significantly higher PD-1 expression was detected in bone marrow CD3+ and CD8+ *T* lymphocytes at diagnosis in the patient group with CD9-negative leukemic blasts. Further studies with a larger patient group may reveal the relationship between CD9 and PD-1 and elucidate the pathophysiological mechanisms. Lower PD-1 expression in patients with CD9 positive leukemic blasts should be evaluated to increase the success rate of CD9 targeted therapies.

AML is a disease caused by impaired differentiation and high proliferation capacity of myeloid hematopoietic progenitor cells in the bone marrow. AML formation and progression begin primarily in the bone marrow and depend on the bone marrow microenvironment [21]. Therefore, it is important to determine the structural characteristics of both leukemic cells and T cells mediate the immune response in the bone marrow. In a study by Jia et al., PD-1 expression in T cells obtained from the bone marrow and peripheral blood was evaluated in newly diagnosed AML patients, and PD-1 expression was found to be higher in the CD8+ *T* lymphocyte subgroup in the bone marrow [8]. The PD-1 receptor has been shown to be a key mediator for “T cell exhaustion,” a state of T cell dysfunction in which the response to persistent antigen stimulation decreases. Based on this finding, Jia et al. stated that the higher level of PD-1 expression on CD8+ *T* cells in the bone marrow of AML patients indicates that there is a suppressive environment in the bone marrow and T cell depletion is higher in these patients. In addition, when they applied functional studies to evaluate cytokine production, proliferation ability and killing capacity, they showed that T lymphocytes in the bone marrow were less functional than those in peripheral blood. These data show that leukemia-reactive CD8+ *T* lymphocytes in the bone marrow are composed of PD-1 positive cells at a higher frequency and are more dysfunctional than lymphocytes in the peripheral blood. This finding provides a strong rationale for therapeutic strategies targeting PD-1 inhibition to enhance the anti-leukemic response in patients with AML. In our study, we evaluated both bone marrow and peripheral blood samples obtained from patients at the time of diagnosis and assessed the difference in PD-1 expression between T lymphocytes associated with the tumor microenvironment in the bone marrow and T lymphocytes circulating in the peripheral blood. The percentage of PD-1 expression in CD3+ and CD8+ *T* lymphocytes in bone marrow samples at the time of diagnosis was significantly higher than that in T lymphocytes in peripheral blood. Consistent with previous studies, this finding supports the idea that PD-1 expression is increased in T lymphocytes associated with the tumor microenvironment in the bone marrow.

In the study by Chen et al., PD-1 expression levels were found to be higher in newly diagnosed or relapsed AML patients compared to healthy controls and it was reported that this may mediate the escape of leukemic cells from the immune system [12]. In a study by Tan et al. PD-1 expression levels in CD4+ and CD8+ *T* lymphocytes were higher than those in healthy volunteers [22]. In our study, the percentage and intensity (MFI) of PD-1 expression in CD3+, CD4+ and CD8+ *T* lymphocytes in peripheral blood samples obtained from patients at the time of diagnosis were found to be significantly higher in all groups than in age- and sex-matched healthy volunteers. This may be considered a response to leukemia in patients. The fact that there was a control group in our study and the expression levels in patients were found to be higher than those in the control group was evaluated as a significant finding. The fact that PD-1 expression levels were higher in patients with AML than in healthy controls suggests that patients may be candidates for treatment with monoclonal antibodies developed against PD-1.

The findings from our study significantly contribute to addressing the primary research question regarding the relationship between PD-1/PD-L1 expression levels and prognosis in acute myeloid leukemia (AML) patients. Our results demonstrated that higher PD-1 expression is associated with a poor treatment response, suggesting that these markers may reflect T-cell dysfunction, which could affect patient prognosis. Despite elevated PD-L1 expression in leukemic blasts, our analysis revealed no significant correlation with treatment response or MRD status. These insights contribute to a more nuanced understanding of the immune landscape of AML and its potential implications in patient prognosis.

The most important limitation of this study was its small sample size. Statistically significant results can be obtained using additional patient data. Some values were borderline significant and may have reached statistically significant levels with an adequate sample size. In addition to PD-1 and PD-L1, co-expression levels of other immune checkpoint markers such as Tim-3/Gal-9, Lag-3, and TIGIT, may reveal the relationship between the immune system and disease. The role of lymphocytes in immune escape can be better understood by determining the expression of these markers in regulatory T cells in addition to CD4 and CD8 T lymphocytes.

## 5 Conclusion

In this study, we aimed to elucidate the association between PD-1 and PD-L1 expression and the prognosis of patients with acute myeloid leukemia in this study. These results support the potential prognostic significance of immune checkpoint markers and reveal the association between PD-1/PD-L1 expression and diverse clinical and phenotypic factors in patients with de novo AML. However, further comprehensive large-scale studies are required in this field.

## Funding

This study was supported by the Ankara University Scientific Research Projects Coordination Office under the project code TTU-2022-2464.

## Data access statement

The Data supporting the findings of this study are available from the corresponding author, Selami Koçak Toprak, upon reasonable request.

## Declaration of generative AI and AI-assisted technologies in the writing process

During the preparation of this work, the authors used Paperpal to edit and format the text. After using this tool/service, the authors reviewed and edited the content as needed and take full responsibility for the content of the publication.

## CRediT authorship contribution statement

**Ugur Calis:** Writing – review & editing, Writing – original draft, Visualization, Validation, Methodology, Investigation, Funding acquisition, Formal analysis, Data curation, Conceptualization. **Merve Aydogan:** Writing – review & editing, Writing – original draft, Methodology, Investigation, Data curation, Conceptualization. **Guldane Cengiz Seval:** Writing – review & editing, Writing – original draft, Supervision, Project administration, Methodology, Investigation, Data curation, Conceptualization. **Klara Dalva:** Writing – review & editing, Methodology, Formal analysis, Conceptualization. **Selami Kocak Toprak:** Writing – review & editing, Writing – original draft, Visualization, Validation, Supervision, Project administration, Methodology, Investigation, Data curation, Conceptualization.

## Declaration of competing interest

The authors declare that they have no affiliations with or involvement in any organization or entity with any financial interest in the subject matter or materials discussed in this manuscript.
